# Spatial and Temporal Dynamics of Pacific Oyster Hemolymph Microbiota across Multiple Scales

**DOI:** 10.3389/fmicb.2016.01367

**Published:** 2016-08-31

**Authors:** Ana Lokmer, M. Anouk Goedknegt, David W. Thieltges, Dario Fiorentino, Sven Kuenzel, John F. Baines, K. Mathias Wegner

**Affiliations:** ^1^Coastal Ecology, Wadden Sea Station Sylt, Alfred Wegener Institute - Helmholtz Centre for Polar and Marine ResearchList auf Sylt, Germany; ^2^Department of Coastal Systems, Royal Netherlands Institute for Sea Research, Utrecht UniversityTexel, Netherlands; ^3^Max Planck Institute for Evolutionary BiologyPlön, Germany; ^4^Institute for Experimental Medicine, Christian-Albrechts-Universität zu KielKiel, Germany

**Keywords:** host-associated communities, *Crassostrea gigas*, distance-decay relationship, spatiotemporal patterns, spatiotemporal dynamics, marine invertebrate microbiota, amplicon analysis

## Abstract

Unveiling the factors and processes that shape the dynamics of host associated microbial communities (microbiota) under natural conditions is an important part of understanding and predicting an organism's response to a changing environment. The microbiota is shaped by host (i.e., genetic) factors as well as by the biotic and abiotic environment. Studying natural variation of microbial community composition in multiple host genetic backgrounds across spatial as well as temporal scales represents a means to untangle this complex interplay. Here, we combined a spatially-stratified with a longitudinal sampling scheme within differentiated host genetic backgrounds by reciprocally transplanting Pacific oysters between two sites in the Wadden Sea (Sylt and Texel). To further differentiate contingent site from host genetic effects, we repeatedly sampled the same individuals over a summer season to examine structure, diversity and dynamics of individual hemolymph microbiota following experimental removal of resident microbiota by antibiotic treatment. While a large proportion of microbiome variation could be attributed to immediate environmental conditions, we observed persistent effects of antibiotic treatment and translocation suggesting that hemolymph microbial community dynamics is subject to within-microbiome interactions and host population specific factors. In addition, the analysis of spatial variation revealed that the within-site microenvironmental heterogeneity resulted in high small-scale variability, as opposed to large-scale (between-site) stability. Similarly, considerable within-individual temporal variability was in contrast with the overall temporal stability at the site level. Overall, our longitudinal, spatially-stratified sampling design revealed that variation in hemolymph microbiota is strongly influenced by site and immediate environmental conditions, whereas internal microbiome dynamics and oyster-related factors add to their long-term stability. The combination of small and large scale resolution of spatial and temporal observations therefore represents a crucial but underused tool to study host-associated microbiome dynamics.

## Introduction

Assessing the temporal and spatial stability of microbial communities is vital for understanding and predicting their response to disturbances (Shade et al., 2012) and thus their functioning in a changing environment. This requires knowledge of the underlying disturbance-free community dynamics (Hunt and Ward, 2015). More precisely, it is crucial to identify regular (e.g., daily, seasonal) patterns, normal range of variation in community dynamics, as well as the processes and factors affecting community assembly and structure (Mutshinda et al., 2009; Costello et al., 2012; Nemergut et al., 2013) to establish a baseline against which to measure disturbance effects (Stenuit and Agathos, 2015).

Although controlled repeated-measures experiments in the laboratory (e.g., Shade et al., 2011; Berga et al., 2012; Lokmer and Wegner, 2015) are indispensable for a mechanistic understanding of how environmental factors affect microbial community dynamics, such results may not directly translate to natural conditions, which represent a blend of abiotic and biotic disturbances varying in their intensity, predictability, spatial scale and duration (Bender et al., 1984; Sousa, 1984; Paine et al., 1998; Berga et al., 2012). Studying natural temporal variability of microbial communities represents a valuable complement to controlled experiments, as it provides an opportunity to estimate the effects of known environmental factors and disturbances, as well as to uncover yet unknown and potentially important determinants of community structure and dynamics (Shade et al., 2013; David et al., 2014; Faust et al., 2015). So far, longitudinal studies and time series have helped to elucidate the dynamics of free-living microbial communities ranging from marine sediments (Gobet et al., 2012) and coastal ocean (Gilbert et al., 2012) to soil (Kato et al., 2015) and freshwater habitats (Peura et al., 2015). Similar studies regarding host-associated microbiota are almost exclusively limited to humans, (e.g., Caporaso et al., 2011; David et al., 2014; DiGiulio et al., 2015), and a handful of model organisms (e.g., Fink et al., 2013; Marino et al., 2014). Whereas temporal patterns have been studied in non-model organisms, the focus remains on the population level and the sampling resolution usually coincides with significant host/environment-related shifts: developmental (e.g., Trabal et al., 2012; Hroncova et al., 2015), seasonal (e.g., Zurel et al., 2011; Bjork et al., 2013; Ransome et al., 2014) or abiotic disturbances (e.g., Vega Thurber et al., 2009; Wegner et al., 2013; Tracy et al., 2015). Longitudinal, individual-based, repeated-measure studies remain scarce (but see Pratte et al., 2015; Glasl et al., 2016).

Analogous to longitudinal studies, examining spatial variability and biogeographical patterns over multiple spatial scales, especially if combined with knowledge of environmental gradients, can shed light on relative importance of stochastic and deterministic processes for shaping microbial communities (Green and Bohannan, 2006; Nakaoka et al., 2006; Caruso et al., 2011; Hanson et al., 2012; Borer et al., 2013). One example is the distance-decay relationship, i.e., decreasing similarity between communities with increasing distance, a universal biogeographical pattern that arises through spatially-correlated environmental conditions or through dispersal limitation and has been demonstrated for microbial communities in both marine and terrestrial habitats (Bell, 2010; Martiny et al., 2011; Zinger et al., 2014; Nguyen and Landfald, 2015). Martiny et al. (2011) found that dispersal limitation affected community similarity within salt marshes, whereas environmental factors played more prominent role at regional or continental scale. Conversely, dispersal-related effects in arctic heathland soils were apparent only at larger scales (Hill et al., 2015), illustrating the importance of a particular context for interpretation of the observed patterns.

Spatially stratified sampling strategies can reveal drivers behind the structure and dynamics of free-living microbial communities (e.g., Caruso et al., 2011; Ristova et al., 2015) as well as of host-associated microbiota (Mihaljevic, 2012). In addition to environmental factors (Moro et al., 2011), examining spatial variation can disentangle geographic influences from those of host life stage (Hroncova et al., 2015), genotype and/or diet (Sudakaran et al., 2012; Yatsunenko et al., 2012; Linnenbrink et al., 2013). However, spatial and geographic information has so far primarily served to delineate core microbiomes (e.g., King et al., 2012; Wong et al., 2013; Dishaw et al., 2014) or to differentiate between the microbiomes of closely related species (Zouache et al., 2011; Phillips et al., 2012). This applies especially to marine hosts (e.g., Morrow et al., 2012; Reveillaud et al., 2014; Trabal Fernandez et al., 2014). Studies considering aspects of within-species spatial variation are less common and focused on large-scale differences between environmentally distinct sites (e.g., Trabal et al., 2012; Pierce et al., 2016; Ziegler et al., 2016). Although marine sedentary organisms offer a good opportunity to examine factors and processes shaping dynamics of their associated microbiota over multiple spatial scales using spatially nested designs, this has not been done yet. Including a temporal component into such studies would further improve our understanding of natural microbial community dynamics (e.g., Fortunato et al., 2012; Ransome et al., 2014; Pierce et al., 2016) and thus refine the reference framework for evaluating disturbance effects.

The Pacific oyster (*Crassostrea gigas*) is such a sedentary organism, highly suitable for the combined estimation of spatial and temporal patterns of microbiome assembly. However, site-specific differences in host-associated microbial communities cannot be separated from host factors by studying natural spatial variability only, as hosts at different sites can be adapted or acclimated to their abiotic and biotic environment (Wendling and Wegner, 2015) or differ due to historical reasons, e.g., invasion histories (Moehler et al., 2011). In contrast to vertebrates, microbiota of most other organisms are closely related to environmental microbial communities (Ley et al., 2008) and translocation experiments with algae (Campbell et al., 2015), and sponges (Burgsdorf et al., 2014) indicate that site is a major determinant of microbiome composition. However, similar experiments with oysters suggest that the influence of site and host factors differs between the tissues (Lokmer et al., 2016). Altogether, combining translocation with a survey of spatial and temporal variation represents a relatively simple means to improve our understanding of the dynamics and function of host-associated microbiota in marine sedentary organisms.

One important function of microbiota that directly contributes to host fitness is defense against pathogens (McFall-Ngai et al., 2013). For example, some of the bacteria inhabiting the oyster hemolymph (a tissue with immune function analogous to vertebrate blood) produce antimicrobial compounds, thus preventing colonization by external pathogens and disease (Defer et al., 2013; Desriac et al., 2014). Hemolymph microbiota can also play part in oyster interactions with abiotic environment (i.e., temperature) by quick (hours to days) adjustments in community composition (Lokmer and Wegner, 2015; Lokmer et al., 2016). Despite the openness of oyster circulatory system and high oyster filtration activity, some bacteria such as *Vibrio* spp. persist in the hemolymph in the absence of an environmental source population (e.g., if held in sterile seawater) over a range of environmental conditions and could thus be considered resident (Vasconcelos and Lee, 1972; Lokmer et al., 2016). Presence of other, transient bacteria is strictly dependent on the external source community and thus reflects immediate environmental conditions (Lokmer et al., 2016). Dynamics of resident and transient components of the hemolymph microbiome are thus likely shaped by different processes and factors. However, despite its significance for oyster fitness, our knowledge about the variability and dynamics of hemolymph microbiota under natural conditions is almost exclusively limited to a subset of cultivable and potentially pathogenic bacteria, mostly of the genus *Vibrio* (Garnier et al., 2007; Wendling et al., 2014; Lemire et al., 2015).

**Figure 1:**
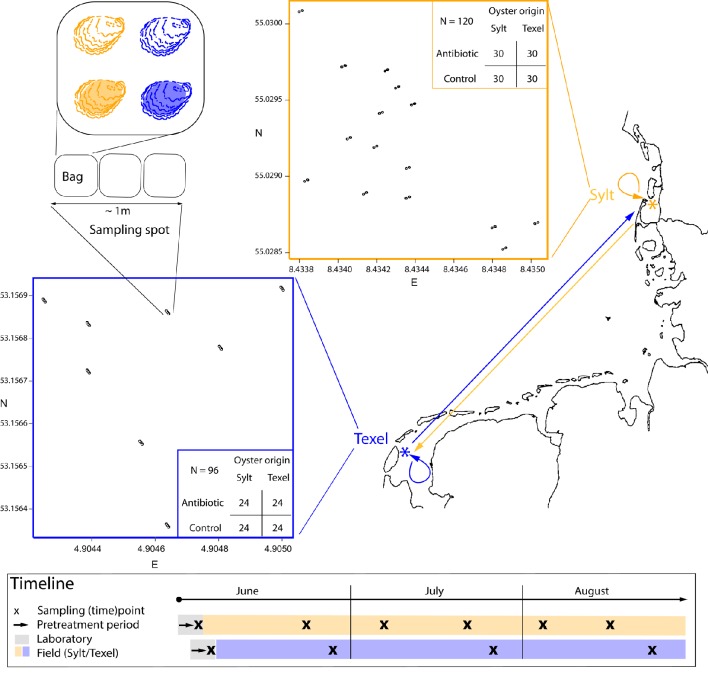
**Experimental design and timeline**. The spatially stratified sampling design is described on the map. The experimental sites (orange = Sylt, blue = Texel) are marked by an asterisk (^*^). The arrows on the map link the oyster collection sites with the deployment sites (i.e., half of the oysters were returned to their collection site while the other half was transplanted). The inserted plots show within-site deployment scheme with all sampling spots. Within each sampling spot there are two (Sylt) or three (Texel) bags with four oysters each. Each oyster in the bag belongs to one of the four treatment groups (i.e., antibiotic treatment/control × Sylt/Texel oyster origin). Note that the colors (orange = Sylt, blue = Texel) can denote site or the oyster origin, depending on the context. Tables show the total and the per treatment sample sizes for each site. The box below the map shows the sampling timeline for both sites.

In order to examine how site and host genotype affect diversity, composition and dynamics of oyster hemolymph microbiota, we performed a reciprocal translocation experiment with two genetically differentiated oyster populations from two sites in the Wadden Sea (Texel, Netherlands and Sylt, Germany, Moehler et al., 2011), and repeatedly sampled hemolymph from the same individuals over one summer season (Figure 1). Prior to the field deployment, we administered antibiotics to half of the oysters in order to remove a large portion of resident microbiota and account for priority effects. In addition, our field deployment (Figure 1) allowed us to examine spatial variation of complete and resident hemolymph microbiota over small (< 1 m) and medium scales (10^1^–10^2^ m, within site). With such spatially and temporally stratified design we can now try to disentangle the relative contribution of different processes (immigration, within-microbiome interactions), and factors (host genetics, geography, environmental conditions) that shape the oyster hemolymph microbiota under natural conditions.

## Materials and methods

The experimental setup and the experiment timeline are shown in Figure 1. The pretreatment, the sampling of the hemolymph and the seawater as well as wet-lab procedures are described in detail in (Lokmer et al., 2016) and therefore only briefly outlined here.

### Oyster collection, laboratory pretreatment and sampling

Oysters from the northern and southern Wadden Sea populations (Moehler et al., 2011) were collected at intertidal mixed oyster/mussel beds at Oddewatt, Sylt, Germany (55° 1′ N, 8° 26′ E) and at de Cocksdorp, Texel, Netherlands (53° 0′ N, 4° 54′ E), respectively. After the removal of epibionts (mainly barnacles) by scrubbing, half of the animals were transported from Sylt to Texel and vice-versa (Figure 1). The laboratory pretreatment was then conducted at the AWI Wadden Sea Station Sylt for the Sylt experiment (i.e., for the oysters later deployed on Sylt) and at the NIOZ Texel for the Texel experiment (i.e., for the oysters later deployed on Texel). For the pretreatment, oysters from each population were divided into two groups: the control group was kept in local 0.2 μm filtered (sterile) seawater, whereas a mix of antibiotics with different mode of action (ampicillin, tetracycline, gentamicin, and kanamycine, Sigma-Aldrich, Hamburg, Germany, final concentration 400 μg/l seawater) was added to the second one to remove as wide range of resident bacteria as possible. After 4 days and prior to the field deployment, hemolymph samples for the analysis of the associated microbial communities were taken with a syringe.

### Field deployment and sampling

Mesh bags with four oysters each (one per treatment combination: origin × antibiotic) were deployed in groups of two (Sylt) or three (Texel) and fixed with iron rods at original sites of collection on Sylt or Texel (Figure 1). In this way, we could estimate how the spatial scale - within the bag groups/sampling spots (< 1 m) and between the sampling spots (10^1^–10^2^ m) - affects hemolymph microbiota. Hemolymph and seawater samples for the analysis of microbiota were taken directly in the field, placed on ice and immediately frozen upon return to the laboratory. Sampling was performed biweekly on Sylt and monthly on Texel. This, along with some other differences between Sylt and Texel (total number of oysters: 120 on Sylt, 96 on Texel; experiment duration: 1 June—23 August 2012 on Sylt, 14 June—24 August 2012 on Texel) was due to logistic reasons.

### DNA extraction, PCR and sequence quality control and preprocessing

DNA was extracted from 200 ± 20 μl of hemolymph with Wizard SV 96 Genomic DNA Purification System (Promega, Manheim Germany) and from the rententate obtained by filtering (0.2 μl) of 100 ml seawater with the DNeasy Blood and Tissue Kit, Qiagen, Hilden, Germany. To check for bacterial contamination of reagents, we included additional blank extractions.

PCR (25 μl, 30 cycles, 1 min annealing at 55°C) of the 16S rRNA gene V1-V2 regions, including positive and negative (ddH_2_O) controls, was performed with equal concentrations of uniquely barcoded 27f and 338r PCR primers (Wang et al., 2015), using 0.5 unit of Phusion Hot Start II High-Fidelity DNA Polymerase per reaction. Equal amounts (estimated by gel electrophoresis and determined fluorometrically) of PCR products were mixed together, purified and sequenced on a Illumina MiSeq platform at the Max Planck Institute for Evolutionary Biology in Plön, Germany.

Sequence quality control and preprocessing was performed as described in Mothur MiSeq SOP (Schloss et al., 2009; Kozich et al., 2013). We defined OTUs (Operational Taxonomic Units) based on a 97% identity threshold. Based on rarefaction curves (not shown), we decided to subsample the dataset to 10,000 reads per sample (6 samples with less than 10,000 were also included, Supplementary Table S1). Due to some oyster mortality, the final dataset comprised of 713 samples in total: 10 seawater, and 703 hemolymph (166 laboratory and 537 field).

Raw demultiplexed reads are deposited at European Nucotide Archive under the study accession number PRJEB9624.

### Statistical analysis

Statistical analyses were performed in R (R Core Team, 2013). The analysis of α-diversity was based on the Shannon's H index, calculated from the complete subsampled dataset (10,000 reads per sample). We first tested for differences between the seawater and oysters within a site and then between the oysters at the two sites using Asymptotic Wilcoxon Mann-Whitney Rank Sum Test (Wilcoxon RS Test). To assess the effects of oyster origin and antibiotic treatment on α-diversity, as well as its temporal dynamics, we fitted a separate linear mixed model for each site with oyster origin, antibiotic treatment, time and their interactions as fixed factors, and with oyster as a random factor. To test if bag and sampling spot influenced α-diversity, we fitted an additional model for each site excluding the laboratory samples, with oyster, bag and sampling spot as random factors. The models were fitted and tested using the packages *lme4* (Bates et al., 2014), *lmerTest* (Kuznetsova et al., 2014), and *MuMIn* (Bartoń, 2014).

In our previous association network analysis of hemolymph microbiota (Lokmer et al., 2016), we identified a cluster consisting of the OTUs abundant in the seawater that could be defined as transient. We analyzed all the hemolymph samples in this study in the same way using the *igraph* package (Csardi and Nepusz, 2006) and again found this transient OTU assemblage (Supplementary Figure S1). To examine how transient microbiota affect β-diversity and distance-decay relationship, we performed the analyses on the complete dataset and excluding the transient OTUs.

For β-diversity, we removed low abundance OTUs (< 10 sequences in the sample) to reduce the dataset complexity (Gobet et al., 2010). The analysis was based on Bray-Curtis distances calculated from hellinger-transformed OTU tables. We used non-metric multidimensional scaling (NMDS) to visualize the overall variability in the dataset (including the seawater samples), and large-scale temporal variability (between sampling points) of hemolymph microbiota. We then analyzed hemolymph communities by constrained analysis of principal coordinates (CAP, Anderson and Willis, 2003), which takes into account only the variability associated with tested predictors, and by Permanova (non-parametric permutational multivariate analysis of variance, Anderson, 2001), using the functions *capscale* resp. *adonis*, both implemented in the *vegan* package (Oksanen et al., 2013). In order to examine how oyster origin, antibiotic treatment and distance affected the β-diversity throughout the summer, we analyzed the hemolymph communities separately at each of the four time-points: before deployment, and once in June, July and August. Although the sampling on Sylt and Texel was not simultaneous, the time difference was at most 10 days and the samples were analyzed together. Two additional sampling points on Sylt were analyzed as well. Variability explained by distance was partialled out prior to plotting CAP results in order to more clearly represent the effects of experimental treatments within sites. To explicitly identify the taxa (from phylum to genus level) contributing to the observed variability, we calculated multivariate generalized (negative binomial) mixed models (GLMs) for each date and for the whole dataset using the *mvabund* package (Wang et al., 2012).

In order to assess bacterial turnover at a large spatiotemporal scale, we calculated average Bray-Curtis distances between the composite communities at different sampling dates (i.e., all samples from a site at a given date were combined into a single sample) as well as the average individual dissimilarity within sampling dates. To estimate how autocorrelation within oyster individuals influenced community structure and dynamics, we compared the average Bray-Curtis distances between all the samples from the same oyster and between the corresponding number of randomly chosen samples from different oysters. To examine within-individual temporal dynamics, we calculated bacterial turnover within oysters as a proportion of OTUs shared between the initial and subsequent sampling points (Gobet et al., 2012).

The distance-decay relationship was analyzed as described in (Martiny et al., 2011). Briefly, we used 1- Bray-Curtis distance as a measure of similarity and calculated all pairwise distances between the samples from the same sampling date. We then calculated linear models for distance-decay relationship including all samples, as well as for within-spot (up to 1 m) and between-spots (tens of meters) distance ranges separately. In order to estimate how this relationship was affected by transient OTUs, we performed the analysis excluding the seawater OTUs and compared the resulting slopes to the original ones.

Temperature is an important determinant of oyster hemolymph microbiota (Lokmer and Wegner, 2015). The mean temperature experienced by the oysters throughout the experiment was estimated from the Sylt seawater temperature time-series (courtesy of Tatyana Romanova, Wadden Sea Station Sylt, Germany) and from NIOZ Jetty, Texel, Netherlands (van Aken, 2008). On Sylt, on-spot fine-scale temperature measurements were taken during sampling to compare microenvironmental and overall temperature variability.

## Results

### Hemolymph microbiota under laboratory and field conditions

**Figure 2:**
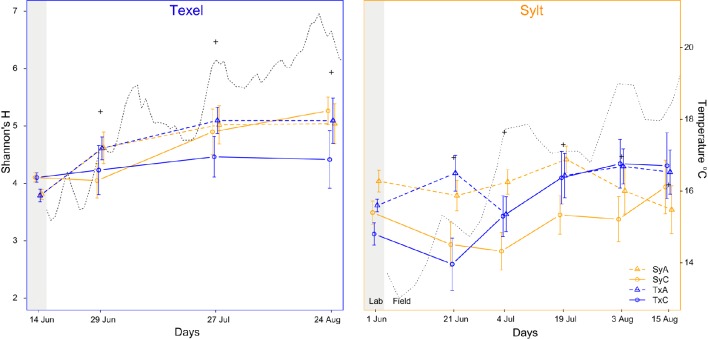
**α-diversity of hemolymph and seawater (+) microbiota on Texel (left, blue frame), and Sylt (right, orange frame) throughout the sampling period**. The dotted line represents mean daily temperature at the sites. Legend explanation: Sy and Tx refer to the oyster origin, A and C to antibiotic treatment and control, respectively.

**Figure 3:**
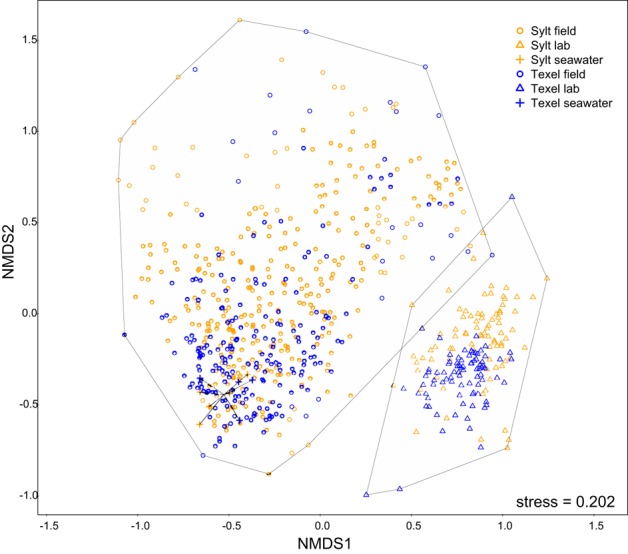
**NMDS plot (Bray-Curtis) depicting variability within the complete dataset (all seawater and hemolymph samples)**. Hulls enclose laboratory and field samples, respectively.

During the pre-treatment, the oysters were kept in the laboratory. As laboratory conditions differ substantially from those in the oyster natural environment, we first examined their effect on the hemolymph microbiota. Whereas we found no systematic difference between α-diversity in the laboratory and in the field (Figure 2), NMDS ordination revealed that the laboratory conditions consistently affected hemolymph community composition at both sites, resulting in a clear separation of laboratory and field samples along the first NMDS axis (Figure 3). Laboratory samples were characterized by higher relative abundances of Fusobacteria, ε- and γ-Proteobacteria (mainly *Arcobacter* and *Vibrionaceae*), whereas α-Proteobacteria, Tenericutes and an unclassified bacterium related to Spirochaetes were more common in the field (Supplementary Figures S2–S4, Supplementary Table S2). In addition, laboratory communities were clearly separated from the seawater samples, which formed a small group within the cluster of field hemolymph communities (Figure 3). Permanova confirmed these results, showing that 13.9% of the compositional variability could be explained by sample type (hemolymph or seawater) for laboratory communities [*F*_(1, 710)_ = 115.241, *p* = 0.001] and only 0.5% for the ones in the field [*F*_(1, 710)_ = 4.503, *p* = 0.001]. This pattern reflects the absence of seawater OTUs in laboratory conditions (i.e., 0.2 μm filtered seawater) thus providing support for their transient character (Supplementary Figure S1), while indicating that these OTUs represent a common and important component of hemolymph microbiota under field conditions. Despite the resemblance between the seawater and hemolymph microbiota in the field, significantly higher heterogeneity of oyster-associated communities [Levene's test for homogeneity of multivariate variances, average distance to median: oyster = 0.588, seawater = 0.407, *F*_(1, 546)_ = 55.9, *p* < 10^−6^, effect size = 0.147, Figure 3], implies that the hemolymph microbiota are not a simple reflection of the microbiota in the surrounding environment, but are shaped by other factors as well.

### Effects of experimental treatments (translocation and antibiotics) on diversity, composition, and dynamics of hemolymph microbiota

To examine the temporal dynamics of α-diversity and quantify the effects of oyster origin and antibiotic treatment, we analyzed each site separately. On Sylt, a strong initial effect of antibiotic treatment persisted for at least 3 weeks (Figure 2A, main effect of treatment and time x treatment interaction of the Sylt model in Table 1). The initial sorting by antibiotic treatment was reversed toward the beginning of July, when diversity in control translocated oysters increased to match the diversity in their antibiotic-treated counterparts (Figure 2A, origin × treatment × time interaction of Sylt model in Table 1). In local Sylt oysters, the difference between the treatment and control remained visible until late July, when the diversity decreased in the antibiotic-treated animals. On Texel, the initial reduction of community diversity following antibiotic treatment was reversed over time in the field (Figure 2B, time × treatment interaction of the Texel model in Table 1). Although Figure 2B indicates a similar trend for Texel as observed on Sylt (i.e., the tendency of translocated oysters to group according to origin), the interactions between origin, antibiotic treatment and time were not significant in the Texel model.

**Table 1:** **Linear mixed models for Shannon's H of hemolymph microbiota on Sylt (dAIC = −6.57, logLik = −673.70 (df = 26), R^2^ marginal = 0.12, R^2^ conditional = 0.34), and Texel (dAIC = −21.92, logLik = −472.13 (df = 18), R^2^ marginal = 0.12, R^2^ conditional = 0.29) during the summer of 2012**.

	**Fixed effects**	**Sum Sq**	**Mean Sq**	***df***	***F***	***p***	**Significant contrasts**	**Estimate**	**2.5% CI**	**97.5%CI**
Sylt	Origin	0.53	0.53	1,115.89	0.43	0.51				
	Treatment	8.90	8.90	1,115.89	7.17	0.01	Antibiotic-Control	0.24	0.06	0.41
	Time	19.65	3.93	5,344.77	3.17	0.01	Linear trend	0.38	0.07	0.69
							Cubic trend	−0.34	−0.62	−0.05
	Origin × Treatment	0.84	0.84	1,115.89	0.68	0.41				
	Origin × Time	7.97	1.59	5,344.77	1.28	0.27				
	Treatment × Time	15.05	3.01	5,344.77	2.43	0.04	(Antibiotic-Treatment) x Linear trend	−0.45	−0.76	−0.14
	Origin × Treatment × Time	11.97	2.39	5,344.77	1.93	0.09	(Sylt-Texel):(Antibiotic-Control):Quadratic trend	−0.27	−0.56	0.02
	*Random effects Oyster*							0.66	0.55	0.88
Texel	Origin	0.50	0.50	1,97.48	0.43	0.52				
	Treatment	1.91	1.91	1,97.48	1.62	0.21				
	Time	40.20	13.40	3,210.12	11.37	0.01	Linear trend	0.76	0.47	1.05
	Origin × Treatment	0.74	0.74	1,97.48	0.62	0.43				
	Origin × Time	1.80	0.60	3,210.12	0.51	0.68				
	Treatment × Time	8.52	2.84	3,210.12	2.41	0.07	(Antibiotic-Treatment) x Quadratic trend	−0.24	−0.51	0.03
	Origin × Treatment × Time	2.50	0.83	3,210.12	0.71	0.55				
	*Random effects Oyster*							0.53	025	0.71

**Figure 4:**
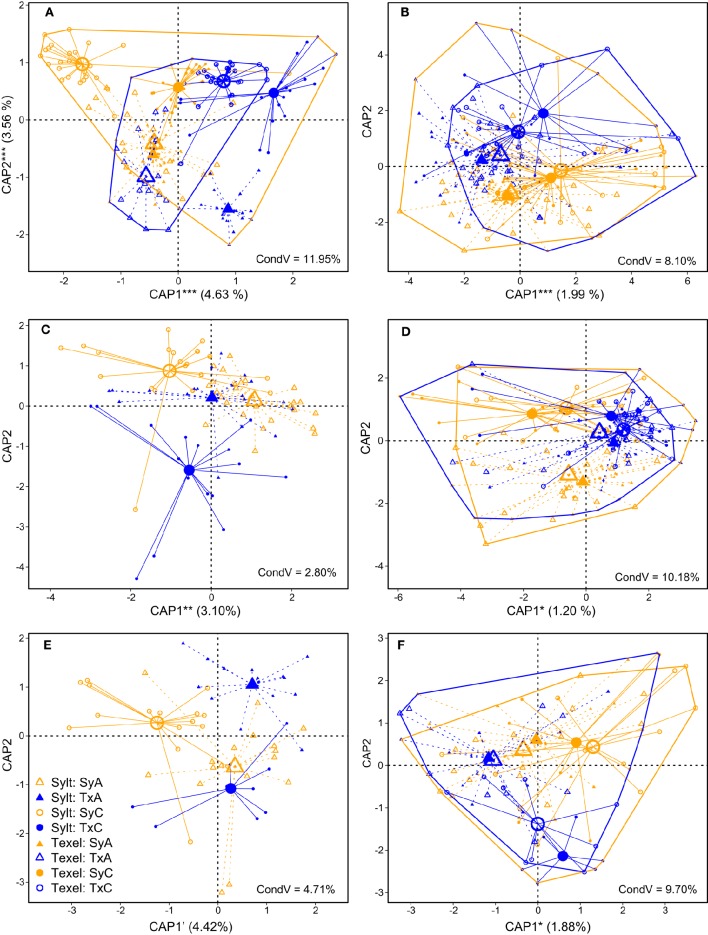
**Constrained analysis of principal coordinates (CAP) of β-diversity of hemolymph communities on Sylt and Texel showing the effects of oyster origin and antibiotic treatment after partialling out the effect of distance (variation explained by distance is given in the plots, “CondV”). (A)** Pre-deployment, **(B)** June, **(C)** July Sylt only, **(D)** July, **(E)** August Sylt only, **(F)** August. The percentages in parentheses represent the variability explained by significant axes. ^***^p < 0.001, ^**^p < 0.01, ^*^p < 0.05, '*p* < 0.1. Hulls enclose all samples from the same site (Sylt = orange, Texel = blue).

To explicitly quantify the effects of antibiotics and oyster origin on hemolymph microbial community composition, we analyzed each sampling point (Figure 1) separately. We observed strong initial effects of oyster origin and antibiotic treatment at both locations (Figure 4A, Table 2). Despite the tendency of the samples to separate according to origin along the first CAP axis and according to antibiotic treatment along the second CAP axis, both Figure 4A and significant two- and three-way interactions between the main factors in the Permanova (Table 2) and multivariate GLMs (Supplementary Table S3) imply that the effects of our treatments depended at least partially on the initial community composition and conditions. Two weeks after deployment, the signature of oyster origin was still apparent, but it disappeared soon afterwards (Table 2, Figures 4B–F). Similarly to α-diversity, the effect of antibiotic treatment persisted for a longer time (i.e., until the end of July). However, the variability explained by oyster origin and antibiotic treatment was generally small (1–2%, Table 2), indicating that the hemolymph community structure was largely determined by other factors (e.g., individual and/or microenvironmental variability). As expected, the exclusion of transient OTUs had virtually no influence on the variability explained by experimental treatments, since their presence and abundance should depend on immediate environmental conditions only (Table 2).

**Table 2:** **Permanova (adonis) showing the effects of oyster origin, antibiotic treatment and distance on hemolymph communities at monthly sampling time points during the experiment**.

**Pre-deployment**	**All OTUs**						**Without transient OTUs**				
	***df***	***SS***	**MS**	**Pseudo F**	***R*^2^**	***p***	***SS***	**MS**	**Pseudo *F***	***R*^2^**	***p***
Origin	1	1.52	1.52	13.09	0.05	0.001	1.49	1.49	12.91	0.05	0.001
Treatment	1	1.25	1.25	10.76	0.04	0.001	1.23	1.23	10.68	0.04	0.001
Site	1	4.71	4.71	40.60	0.16	0.001	4.73	4.73	40.98	0.16	0.001
Origin × Treatment	1	0.70	0.70	6.02	0.02	0.001	0.69	0.69	6.02	0.02	0.001
Origin × Site	1	1.42	1.42	12.28	0.05	0.001	1.41	1.41	12.21	0.05	0.001
Treatment × Site	1	1.35	1.35	11.67	0.05	0.001	1.36	1.36	11.78	0.05	0.001
Origin × Treatment × Site	1	0.42	0.42	3.63	0.01	0.002	0.42	0.42	3.59	0.01	0.001
Residuals	157	18.21	0.12		0.62		18.13	0.12		0.62	
Total	164	29.58			1.00		29.47			1.00	
**JUNE**											
Origin	1	0.38	0.38	1.83	0.01	0.045	0.44	0.44	1.95	0.01	0.026
Treatment	1	0.76	0.76	3.67	0.02	0.002	0.81	0.81	3.56	0.02	0.002
Distance	2	3.87	1.94	9.31	0.10	0.001	3.57	1.79	7.84	0.09	0.001
Origin × Treatment	1	0.15	0.15	0.72	0.00	0.747	0.18	0.18	0.79	0.00	0.677
Origin × Distance	2	0.33	0.17	0.80	0.01	0.731	0.38	0.19	0.84	0.01	0.705
Treatment × Distance	2	0.39	0.19	0.93	0.01	0.518	0.42	0.21	0.92	0.01	0.556
Origin × Treatment × Distance	2	0.48	0.24	1.16	0.01	0.217	0.50	0.25	1.10	0.01	0.295
Residuals	155	32.24	0.21		0.83		35.33	0.23		0.85	
Total	166	38.61			1.00		41.65			1.00	
**SYLT JULY**											
Origin	1	0.30	0.30	1.24	0.01	0.211	0.33	0.33	1.29	0.02	0.173
Treatment	1	0.56	0.56	2.30	0.03	0.016	0.60	0.60	2.35	0.03	0.011
Distance	2	0.61	0.31	1.25	0.03	0.166	0.61	0.30	1.20	0.03	0.176
Origin × Treatment	1	0.39	0.39	1.57	0.02	0.074	0.40	0.40	1.58	0.02	0.070
Origin × Distance	2	0.29	0.15	0.60	0.01	0.980	0.33	0.16	0.64	0.02	0.974
Treatment × Distance	2	0.52	0.26	1.06	0.02	0.359	0.45	0.23	0.89	0.02	0.631
Origin × Treatment × Distance	2	0.68	0.34	1.40	0.03	0.088	0.72	0.36	1.43	0.03	0.064
Residuals	71	17.39	0.24		0.84		18.01	0.25		0.84	
Total	82	20.74			1.00		21.45			1.00	
**JULY**											
Origin	1	0.23	0.23	1.11	0.01	0.291	0.25	0.25	1.13	0.01	0.266
Treatment	1	0.37	0.37	1.78	0.01	0.047	0.36	0.36	1.66	0.01	0.063
Distance	2	3.95	1.98	9.64	0.12	0.001	3.98	1.99	9.11	0.11	0.001
Origin × Treatment	1	0.17	0.17	0.83	0.01	0.636	0.20	0.20	0.89	0.01	0.519
Origin × Distance	2	0.33	0.17	0.81	0.01	0.757	0.38	0.19	0.87	0.01	0.687
Treatment × Distance	2	0.56	0.28	1.38	0.02	0.075	0.58	0.29	1.32	0.02	0.084
Origin × Treatment × Distance	2	0.71	0.36	1.73	0.02	0.018	0.71	0.35	1.62	0.02	0.016
Residuals	131	26.85	0.20		0.81		28.65	0.22		0.82	
Total	142	33.17			1.00		35.10			1.00	
**SYLT AUGUST**											
Origin	1	0.27	0.27	1.08	0.02	0.323	0.29	0.29	1.11	0.02	0.290
Treatment	1	0.26	0.26	1.02	0.02	0.385	0.25	0.25	0.95	0.02	0.513
Distance	2	0.69	0.35	1.36	0.05	0.057	0.72	0.36	1.36	0.05	0.069
Origin × Treatment	1	0.24	0.24	0.95	0.02	0.497	0.26	0.26	0.99	0.02	0.444
Origin × Distance	2	0.40	0.20	0.78	0.03	0.868	0.42	0.21	0.79	0.03	0.868
Treatment × Distance	2	0.64	0.32	1.26	0.04	0.127	0.65	0.33	1.23	0.04	0.135
Origin × Treatment × Distance	2	0.31	0.15	0.61	0.02	0.991	0.33	0.16	0.62	0.02	0.992
Residuals	47	11.96	0.25		0.81		12.44	0.26		0.81	
Total	58	14.77			1.00		15.37			1.00	
**AUGUST**											
Origin	1	0.22	0.22	1.03	0.01	0.349	0.23	0.23	0.98	0.01	0.427
Treatment	1	0.30	0.30	1.37	0.01	0.144	0.24	0.24	1.02	0.01	0.382
Distance	2	2.12	1.06	4.90	0.10	0.001	2.44	1.22	5.23	0.11	0.001
Origin × Treatment	1	0.27	0.27	1.27	0.01	0.18	0.27	0.27	1.16	0.01	0.273
Origin × Distance	2	0.36	0.18	0.82	0.02	0.767	0.39	0.20	0.84	0.02	0.747
Treatment × Distance	2	0.40	0.20	0.93	0.02	0.556	0.40	0.20	0.86	0.02	0.708
Origin × Treatment × Distance	2	0.56	0.28	1.29	0.03	0.132	0.59	0.30	1.27	0.03	0.125
Residuals	74	15.99	0.22		0.79		17.26	0.23		0.79	
Total	85	20.22			1.00		21.83			1.00	

*Samples from Sylt and Texel on comparable sampling points in June, July and August are analyzed together. Two additional sampling points on Sylt, one in July and one in August are analyzed separately*.

### Spatial patterns and dynamics of hemolymph microbiota across scales

At a large scale, α-diversity was higher on Texel than on Sylt, in the hemolymph (Shannon's H median: Texel = 4.422 (*N* = 289), Sylt = 3.816 (*N* = 414); Wilcox RS Test: *Z* = −7.032, *p* < 0.001, effect size = −0.265) as well as in the seawater (Shannon's H median: Texel = 6.186 (*N* = 4), Sylt = 4.468 (*N* = 6); Wilcox RS Test: *Z* = −2.559, *p* = 0.011, effect size = −0.810). Within-site analysis revealed lower diversity in the hemolymph compared to the seawater on Texel (Wilcoxon RS Test: *Z* = −2.335, *p* = 0.020, effect size = −0.127), and no differences on Sylt (Wilcoxon RS Test: *Z* = −0.759, *p* = 0.448). Similarly, we found a positive correlation between the seawater temperature and the diversity of the hemolymph microbiota on Texel (Kendall's τ = 0.140 ± 0.032, *p* = 0.01), but not on Sylt (*p* = 0.388). These discrepancies between the two sites suggest that α-diversity may be influenced by different biotic and abiotic factors at each site. To test for fine-scale spatial influence, we included the bag and the sampling spot as random factors in the α-diversity field-only models (Supplementary Table S4). However, we found no evidence that the spatial proximity resulted in more similar diversity values (Supplementary Table S4, random effects for spot and bag).

Regarding β-diversity, a relatively high amount of variability was explained by distance in all except Sylt-only models (Table 2 and conditional variability in Figure 4), indicating that this distance-related variability primarily reflected the differences between the sites. Multivariate GLMs (Supplementary Tables S5–S8) confirmed the considerable influence of site on community composition. However, the majority of significantly differing taxa were not very abundant (except for the unclassified Spirochaetes-related bacteria; compare univariate significant scores in Supplementary Tables S5–S8 with the taxa depicted in Supplementary Figures S2–S4, where only the taxa with >0.1 relative abundance in at least one oyster group are shown). These rare taxa may thus represent transient microbiota that reflect site-specific environmental conditions, as consistently higher abundance of Cyanobacteria on Sylt or *Oceanospirillaceae* on Texel might suggest (Supplementary Tables S5–S7).

Despite the differences between Texel and Sylt, the average dissimilarity between the individuals within a site largely exceeded overall dissimilarity between the sites (pairwise Bray-Curtis distance - mean ± SD - between individuals within a site on the same sampling date: Sylt = 0.779 ± 0.105, Texel = 0.751 ± 0.110; between Sylt and Texel composite communities on the same date = 0.490 ± 0.047). This high between-individual variability might suggest the important role of factors such as oyster genotype, physiology, condition and microenvironmental heterogeneity.

**Figure 5:**
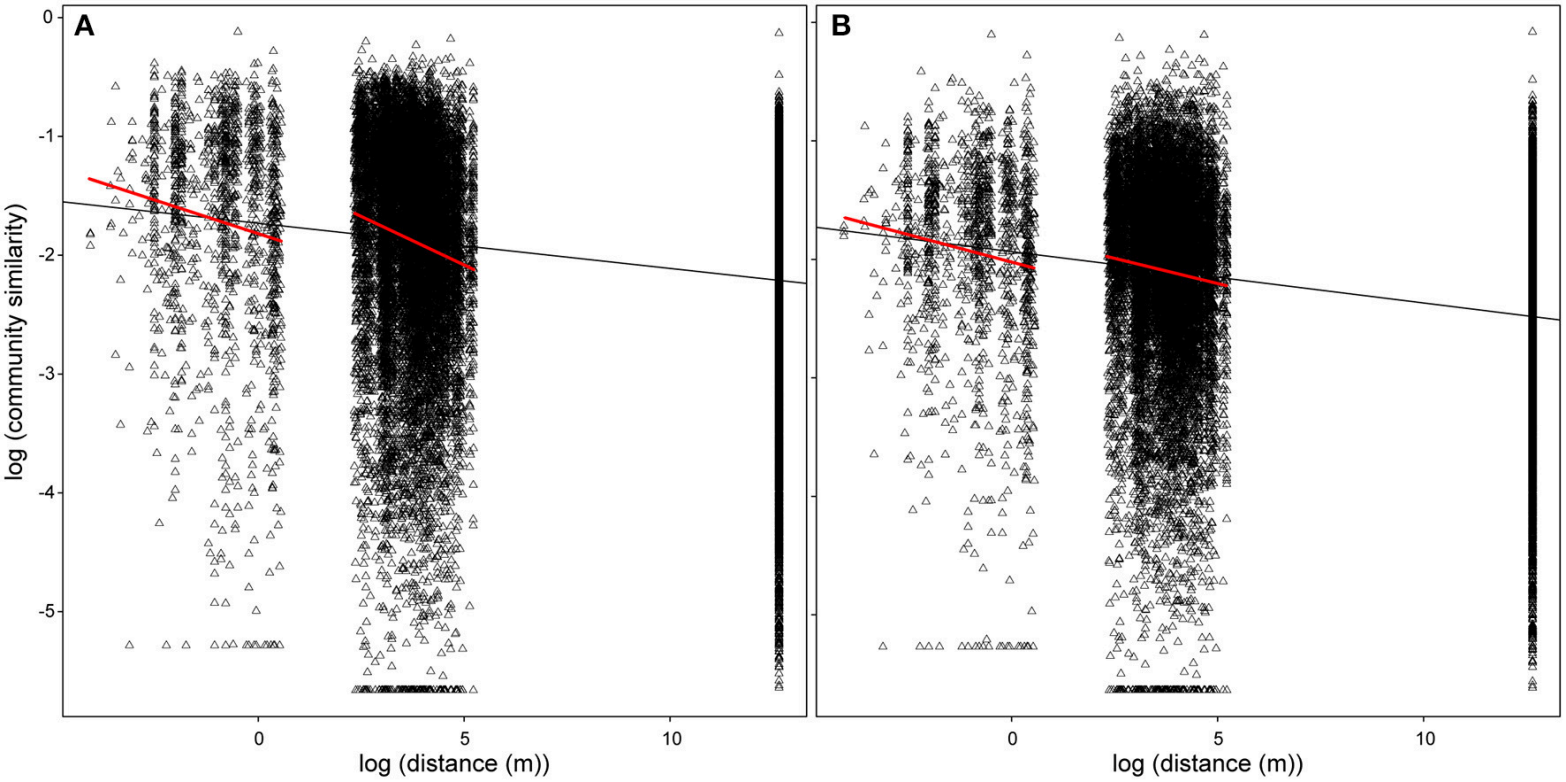
**Distance-decay relationship including (A) all OTUs and (B) resident (excluding seawater) OTUs**. The lines represent linear models fitted for the given spatial scale.

**Figure 6:**
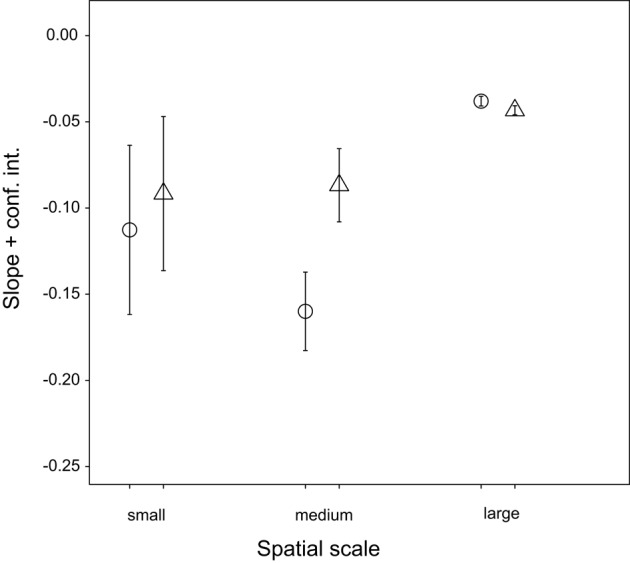
**Effect of transient OTUs on slope of distance-decay relationship on small, medium and overall spatial scale**. ○ = all OTUs, △ = without transient.

Although the community composition varied widely at each spatial scale, we detected a negative correlation between community similarity and geographic distance (Figure 5, overall distance-decay slope: *b* = −0.024, *p* < 0.001). The relationship was significantly stronger over small (up to 1 m, within spot: *b* = −0.102, *p* < 0.001) and intermediate (between spots, up to 186 m: *b* = −0.113, *p* < 0.001) spatial scales. Exclusion of transient OTUs affected neither overall (Figure 6, *b* = −0.027, *p* < 0.001) nor the small-scale distance-decay relationship (*b* = −0.082, *p* < 0.001). On the other hand, it flattened the distance-decay slope at intermediate spatial scale (Figure 6, *b* = −0.034, *p* < 0.001), suggesting that the transient OTUs could indeed reflect the higher probability of adjacent spots to experience similar environmental conditions during immersion. However, the same analysis performed for each month separately revealed that the distance-decay relationship varied over time (Supplementary Figure S4). The overall results were driven by patterns observed in June and July, whereas in August both large- and intermediate-scale slopes were steeper if transient OTUs were excluded. In fact, in August we found no significant distance-decay relationship at intermediate scale with the transient OTUs included (*p* = 0.065) or at the small scale regardless of the microbial community portion considered (complete: *p* = 0.167, without transient: *p* = 0.169).

### Temporal dynamics of hemolymph microbiota: sites and individuals

Overall, α-diversity increased over the course of the experiment, with a clearer and more pronounced effect on Texel (Figure 2, Table 1, main effect of time in both Sylt and Texel model). Including the oyster individual as a random effect into the α-diversity models substantially increased the amount of explained variability, as illustrated by the difference between marginal (fixed effects only) and conditional (including random factors) R^2^ in the legend to Table 1. Thus, the factors like oyster genotype, but also historical contingency might play an important role in shaping the within-diversity of hemolymph microbiota.

**Figure 7:**
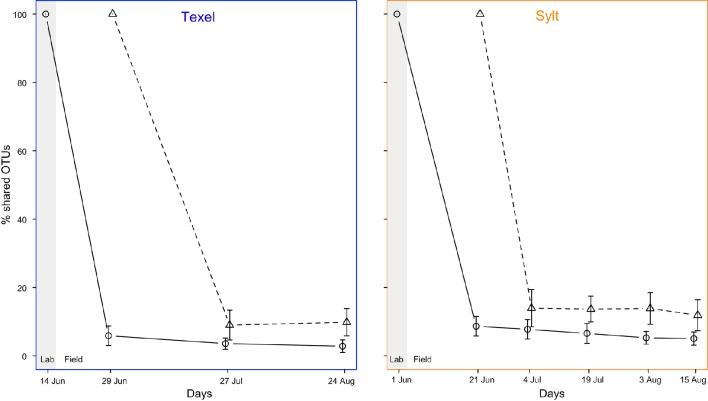
**Bacterial community turnover, i.e., the percentage of OTUs shared between the samples from the same individual at the initial and the subsequent sampling points (○ = all samples, △ = laboratory samples excluded)**.

Similar to spatial patterns, the analysis of large-scale temporal dynamics of β-diversity revealed relatively high degree of stability over the examined period (mean ± SD Bray-Curtis distance between composite communities on individual sampling dates: Sylt = 0.425 ± 0.060, Texel = 0.447 ± 0.068), with NMDS plot showing a slight tendency of hemolymph communities sampled in August to cluster separately from the rest at both sites (Supplementary Figure S5). However, the August samples were indistinguishable from the June and July samples down to the genus level (Supplementary Figure S3), indicating that the shift occurred at the OTU level. This overall temporal stability likely resulted from relatively stable overall environmental conditions throughout the sampling period (Supplementary Figure S6). On the other hand, temporal variability at the individual scale was substantial (mean ± SD Bray-Curtis distance = 0.818 ± 0.140), probably reflecting highly dynamic microenvironmental conditions experienced by the oysters at short timescales. Still, albeit high, the within-oyster temporal variability was smaller than the among-oyster variation (mean ± SD Bray-Curtis distance among oysters = 0.876 ± 0.105, Wilcox RS test: *Z* = −9.283, *p* < 10^−6^), implying that the oyster-related factors such as genotype as well as internal community dynamics and priority effects shape the composition of the hemolymph microbiota. In addition, high but constant within-individual turnover rate between consecutive sampling times suggests non-directional fluctuations in community composition during the sampling period, i.e., repeated occurrence of at least some taxa (Figure 7).

## Discussion

Animal fitness is inextricably linked to the stability of its associated microbiota. An essential prerequisite for assessing the stability is a thorough understanding of factors and processes shaping the microbial community structure and dynamics. To identify these and their relative importance under natural conditions can be challenging, especially in a highly variable environment, such as the intertidal. Here, we combined experimental manipulation with a field survey of temporal and spatial patterns in Pacific oyster hemolymph microbiota over multiple scales to improve our understanding of their dynamics in complex natural environments. Our observations revealed high small-scale spatial variability in the field. This, together with pronounced differences between microbial community composition in the laboratory and in the field, implies a quick response of the hemolymph microbiota to large shifts in environmental conditions. Although environmentally-dependent acquisition/loss of transient microbiota indicate an important role of colonization by exogenous microbes, the persistent effects of translocation and antibiotic treatment together with recognizable individual temporal dynamics suggest that the community structure and dynamics are also influenced by host-related factors as well as by biotic interactions within the microbiome.

### Resident and transient hemolymph microbiota under laboratory and natural conditions

Although common in oyster hemolymph under field conditions, seawater OTUs were virtually absent from the hemolymph-associated communities in the laboratory, where the oysters were kept in the filtered seawater. Dependence on the environmental source population implies that these OTUs could be considered transient (Vasconcelos and Lee, 1972). As such, they are expected to strongly depend on the immediate environmental conditions, in this case immersion during the tidal cycle in the field (Lokmer et al., 2016) and thus should not (and did not) reflect the long-term effects of our experimental treatments (Table 2, compare the variability explained by the treatments including/excluding transient microbiota). In addition, the strong effect of transient microbiota on the distance-decay slope over microenvironmentally variable scales, i.e., between the sampling spots (Figure 5), provides further support for the link between the immersion and the dynamics of transient bacteria. Explicitly, whereas the abiotic conditions experienced by oysters are very similar within the sampling spots (< 1 m), the factors such as immersion time and tidal currents likely differ between the spots in a distance-dependent manner, subsequently affecting the dynamics of the transient microbiota and the distance-decay slope.

Whereas our results clearly support the classification of seawater OTUs as transient, it is less clear which bacteria should be considered resident. For example, an unindentified bacterium close to Spirochaetes and a Tenericutes OTU were both abundant in the field and rare in the laboratory, indicating they might be transient. However, these bacteria have been previously found in Pacific oysters in Tasmania (Fernandez-Piquer et al., 2012), suggesting their affinity to form associations with oysters. Both Spirochaetes and Tenericutes are commonly isolated from various oyster tissues (Prieur et al., 1990; Green and Barnes, 2010; Husmann et al., 2010; King et al., 2012; Trabal et al., 2012; Wegner et al., 2013; Trabal Fernandez et al., 2014; Lokmer et al., 2016). However, Spirochaetes and Tenericutes are rare in the hemolymph in the laboratory (Lokmer and Wegner, 2015; Lokmer et al., 2016), where high abundance of Tenericutes is mainly linked to stress or even mortality (Lokmer and Wegner, 2015). Increased abundance of Tenericutes in the field observed here could thus have been due to a secondary infection of the injection site on the adductor muscle caused by repeated hemolymph sampling (Ayling et al., 2011). On the other hand, potential benefits that Tenericutes and Spirochaetes provide to their hosts would likely be nutrition-related (Prieur et al., 1990; Tanaka et al., 2004; Fraune and Zimmer, 2008) and the decrease in their abundance in the laboratory might have been linked to starvation, as oysters were not fed during the pretreatment period (Green and Barnes, 2010). Similarly, high abundance of *Vibrio* and *Arcobacter* commonly found in the laboratory (Lokmer and Wegner, 2015; Lokmer et al., 2016) could result from stationary conditions and represent a pre-disease state (Petton et al., 2015a). However, these bacteria are also commonly isolated in the field (Garnier et al., 2007; Wendling et al., 2014; Lokmer et al., 2016) and may play role in pathogen defense and acclimation (Defer et al., 2013; Lokmer and Wegner, 2015). Due to geographical (Petton et al., 2015b) and seasonal (Wegner et al., 2013; Wendling et al., 2014; Pierce et al., 2016) differences in dynamics of these potential hemolymph residents, further studies addressing large-scale spatial and temporal variation of hemolymph microbiota, their function as well as the factors affecting their interactions with oysters are needed (Pruzzo et al., 2005; Aagesen et al., 2013).

### Variation of oyster microbiota related to origin and antibiotic treatment

Weeks-lasting effects of antibiotic treatment and oyster origin imply gradual turnover of resident microbiota and demonstrate the existence of internal community dynamics and importance of historical contingencies (Nemergut et al., 2013). Although antibiotics can have long-term negative effects on diversity in some cases (Stein et al., 2013), they increase diversity in others, probably due to the increased invasion susceptibility of the affected communities (Shea et al., 2004; Robinson et al., 2010). In relatively stable and isolated environments, such as mammalian gut, antibiotic-induced changes may induce permanent shifts resulting in alternative stable states (Stein et al., 2013). Oyster hemolymph, on the other hand, is a highly variable habitat closely connected with the external environment, and thus the establishment of such stable states in the associated microbial community is highly unlikely.

Genetic differentiation between oysters from Texel and Sylt (Moehler et al., 2011) could have contributed to the observed differences in β-diversity since oyster microbiota can assemble according to host genotype (Wegner et al., 2013). In addition, many *Vibrio* spp. are pathogenic and oyster populations rapidly adapt to their local *Vibrio* species (Rosa et al., 2012; Wendling and Wegner, 2015), suggesting that at least parts of hemolymph microbiota are affected by host genotype. However, the gradually decreasing difference between translocated and local oysters at both sites implies that the divergence in community composition on the host origin level was in the long run mainly affected by site, a common pattern found in marine sedentary animals (Burgsdorf et al., 2014; Lear et al., 2014; Campbell et al., 2015). Gradual turnover of resident bacteria following translocation has previously been demonstrated for *Vibrio* spp. populations in the oyster hemolymph (Wendling et al., 2014). Interestingly, the diversity of hemolymph microbial communities in control translocated oysters did not initially differ from their local counterparts, but increased during July at both sites, matching the timespan reported for the *Vibrio* spp. turnover (Wendling et al., 2014). This effect was only marginally significant, but it is tempting to speculate that this trend might have been linked to inability of translocated-oyster immune system to control the resident microbiota acquired from the new environment, resulting in more bacteria evading the oyster immune defenses and establishing in the hemolymph.

Close contact of the hemolymph with the external environment might dampen genotype-specific community assembly as opposed to other tissues more shielded from the environment (Wegner et al., 2013; Lokmer et al., 2016) as well as prevent the evolution of specialist hemolymph symbionts (Preheim et al., 2011). Nevertheless, the seawater and the coastal sediments are characterized by seasonally recurring bacterial populations (Gilbert et al., 2012; Gobet et al., 2012), likely resulting in predictable encounters between the oysters, their resident bacteria and external microbiota. Unpredictable disturbances to any component of that system, such as translocation or antibiotic treatment here, might influence the community structure and dynamics and subsequently affect oyster fitness (Lokmer et al., 2016).

### Hemolymph microbiota across temporal and spatial scales

At coarse temporal and spatial resolution, the hemolymph microbiota appeared relatively stable throughout the sampling period and we found little variation in dominant taxa (Supplemetary Figures S2, S3, Supplementary Tables S2, S5–S7). Previous studies have demonstrated a strong effect of temperature on the hemolymph microbiota (Lokmer and Wegner, 2015), but have also shown that the community structure in the natural conditions exhibits a seasonal pattern and does not respond to quick temperature shifts (Wendling et al., 2014). In addition, we can show that hemolymph microbiota are also affected by the immediate external microbial environment (Lokmer et al., 2016). Therefore, high large-scale spatiotemporal stability can be explained by stable mean temperature throughout the sampling period at both sites (Figure 2, Supplementary Figure S6) as well as by seasonally stable microbial communities in oyster surroundings, namely in the sediments and the seawater (Campbell et al., 2011; Gilbert et al., 2012; Gobet et al., 2012).

Nevertheless, we detected differences, albeit rather small and mostly constrained to less abundant phylotypes (Supplementary Tables S5–S7), between the sites in both diversity and composition. Apart from dispersal limitation, regarding primarily OTUs within the dominant lineages, these differences might have been related to environmental factors such as smaller sediment grain size at Texel, which could affect the structure of associated microbial communities (Jackson and Weeks, 2008; Legg et al., 2012) in the environment and also result in higher number of suspended particles in the seawater with consequences for the oyster filtering activity (Riisgard, 1988; Frechette et al., 2016) and microbiota.

However, it is important to remember that, although the 16S rRNA gene and its fragments represent an important tool for understanding of microbial communities, they lack resolution power. In addition, taxonomy is only partially consistent with ecology (Koeppel and Wu, 2012) and allows solely for distinction between broad habitat types (Schmidt et al., 2014). 16S rRNA defined OTUs may consist of variety of ecotypes, and, in case of host-associated bacteria, they can significantly differ in crucial traits such as virulence (Koeppel and Wu, 2013; Lemire et al., 2015; Wendling and Wegner, 2015). Moreover, closely related bacteria exhibit adaptation at very small spatial scales (Belotte et al., 2003). Therefore, although the communities at both sites and throughout the summer appear similar through the lens of 16S rDNA based taxonomy, they can actually consist of ecologically different bacteria with important consequences for hemolymph microbiota dynamics and their oyster hosts.

Interestingly, in August we observed both a slight shift in community composition as well as a change of the distance-decay relationship. It remains unclear whether there is a link between the both, but the observed changes might have been related to spawning, which occurred during August at both sites (according to observations of spat size in autumn). Spawning represents a stressful period in the oyster lifecycle, increasing the susceptibility to pathogens and affecting the composition of associated *Vibrio* communities (Wendling et al., 2014) and likely of other oyster-associated bacterial populations.

In contrast to large-scale stability, the within-individual temporal variability and the between-individual variability at small spatial scale were high, likely reflecting microenvironmental spatiotemporal heterogeneity. Virtual lack of directionality in large-scale dynamics in combination with this high small-scale variability suggests that the latter could be related to extreme but periodic environmental fluctuations in the intertidal. Namely, the quick response of the hemolymph microbiota to such fluctuations (Lokmer and Wegner, 2015; Lokmer et al., 2016) may result in pronounced but predictable (cyclic) dynamics, as bacteria disappear from the hemolymph or fall below the detection limit (Caporaso et al., 2012; Shade et al., 2013, 2014), and re-colonize the oyster or increase in abundance when the conditions are right again. High but constant turnover rate (Figure 6) indeed suggests that bacterial populations may disappear or become very rare, but reappear at a later stage (Gobet et al., 2012). In addition to strong influence of the environment, factors such as oyster genotype, physiology and health condition are also likely to affect the structure of the hemolymph microbiome (Wegner et al., 2013; Lokmer and Wegner, 2015), accounting for the pronounced differences between the oysters and at the same time for the consistent temporal dynamics within individuals (i.e., large portion of α-diversity variability explained by individual).

Overall, our results confirm that temporal (Shade et al., 2013) and spatial (Martiny et al., 2011; Borer et al., 2013; O'Brien et al., 2016) scale strongly affect inference about community stability and dynamics (Faust et al., 2015). High perceived temporal stability of microbiota associated with the subtidal sessile marine invertebrates (Erwin et al., 2012; Bjork et al., 2013; Pita et al., 2013; Hardoim and Costa, 2014) is often related be low temporal and/or taxonomic resolution. While the analysis of composite communities reveals influences of large-scale environmental and host factors (e.g., site, season, tissue type), focusing on small-scale individual dynamics is necessary for deciphering host-microbiota interactions and thus for understanding their role for animal survival.

## Conclusion

Our study aimed to examine the dynamics of Pacific oyster hemolymph microbiota under natural conditions. By switching the focus between large- and small-scale temporal and spatial variation, we identified potentially important factors and processes shaping the hemolymph microbiome. High small-scale variability (within-site or within-individual) likely reflects microenvironmental heterogeneity as well as host genetic differences, with the range of variability determined by large-scale mean abiotic conditions and internal microbiome interactions.

As drivers of host-associated community dynamics are numerous and act in a scale-dependent manner, the appropriate scale for investigations depends on the questions that one aims to address. Spatially stratified sampling designs and the analysis of individual and population temporal dynamics provide useful hints for choosing the adequate resolution. In this way one can also design experiments that will more closely mimic characteristics of the natural environment crucial for dynamics and assembly of host-associated microbiota and thus contribute to elucidating their role for animal fitness.

## Author contributions

AL planned and conducted the experiment, collected and analyzed the data and wrote the manuscript. AG and DT collected the data and critically revised the manuscript. DF took part in the analysis and interpretation of the data and critically revised the manuscript, SK collected the data, JB critically revised the manuscript, KW planned the experiment and wrote the manuscript.

## Funding

This study was supported by the DFG (Deutsche Forschungsgemeinschaft) Emmy Noether Programme (We4641/1-3), Excellence Cluster 306 “Inflammation at Interfaces,” the Netherlands Organization for Scientific Research (NWO) and the German Federal Ministry of Education and Research (BMBF, NWO-ZKO project 839.11.002) and the International Max Research School for Evolutionary Biology. All procedures were performed according to national and European law and experiments were approved by the local authorities.

### Conflict of interest statement

The authors declare that the research was conducted in the absence of any commercial or financial relationships that could be construed as a potential conflict of interest.
