# Supplementary material for: Spatial and Temporal Dynamics of Pacific Oyster Hemolymph Microbiota across Multiple Scales
Source: Front Microbiol. 2016 Aug 31;7:1367. doi: 10.3389/fmicb.2016.01367 (PMC5006416; doi:10.3389/fmicb.2016.01367)
Supplement: Supplementary file 2 [file Image1.pdf]

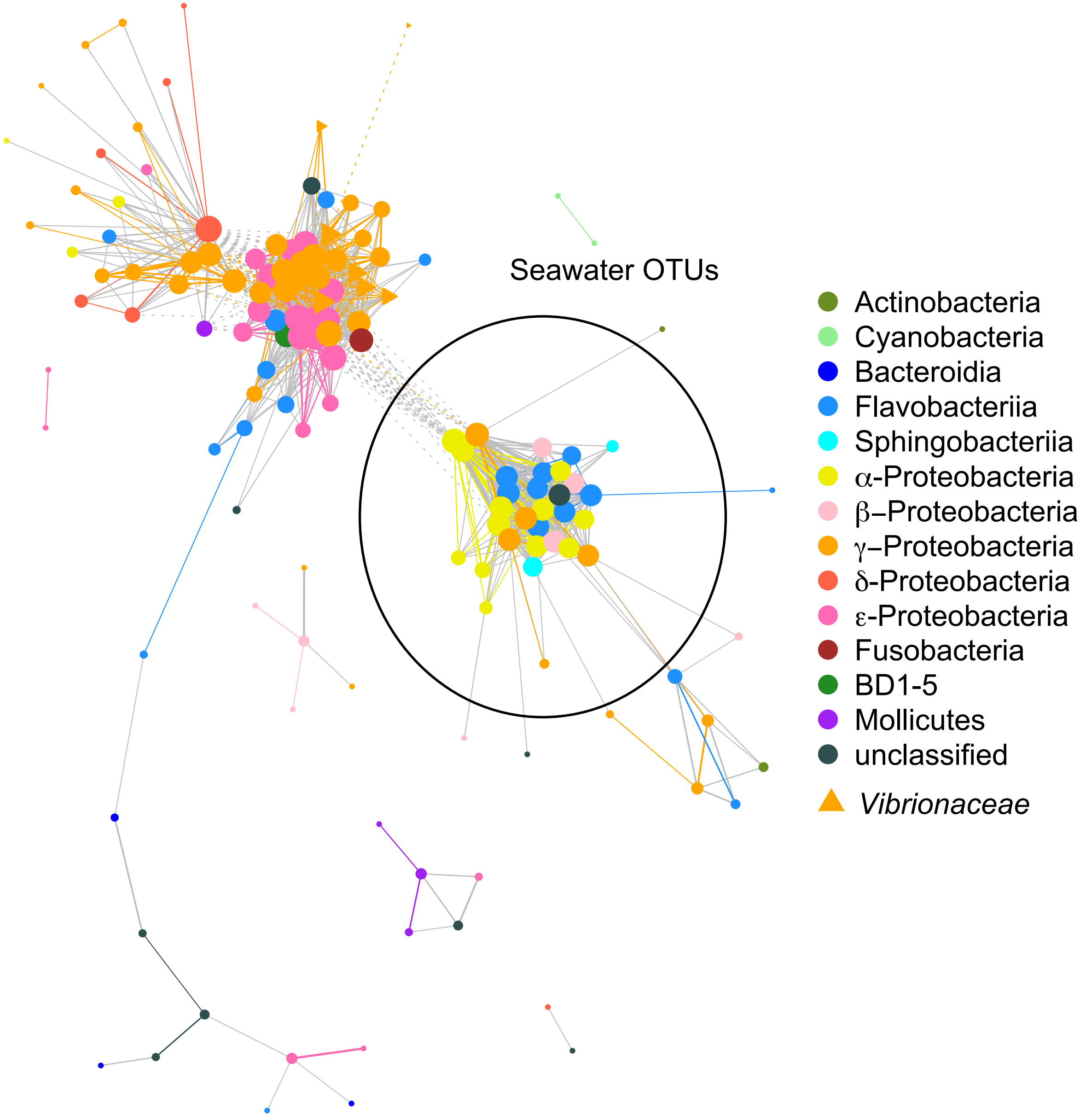


Supplementary Figure S1. Association network of hemolymph microbiota based on all samples. The excluded transient, i.e. seawater OTUs are enclosed in a circle.

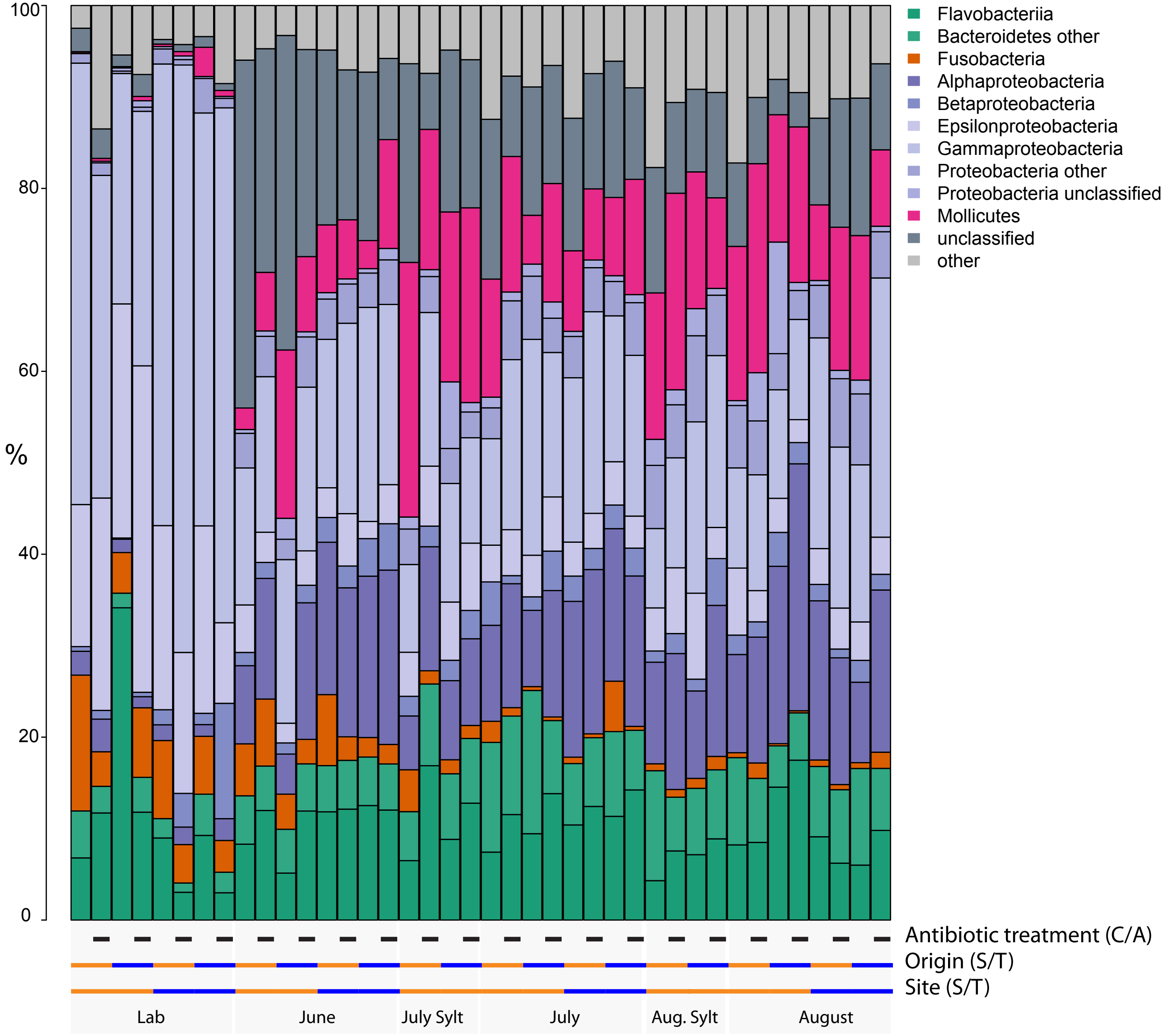


Supplementary Figure S2. Class level community structure of oyster hemolymph microbiota grouped by date, site, origin and antibiotic treatment. Only taxa with > 0.1 relative abundance are shown. The "unclassifed" bacteria shown in grey mainly consist of a single OTU related to Spirochaetes.

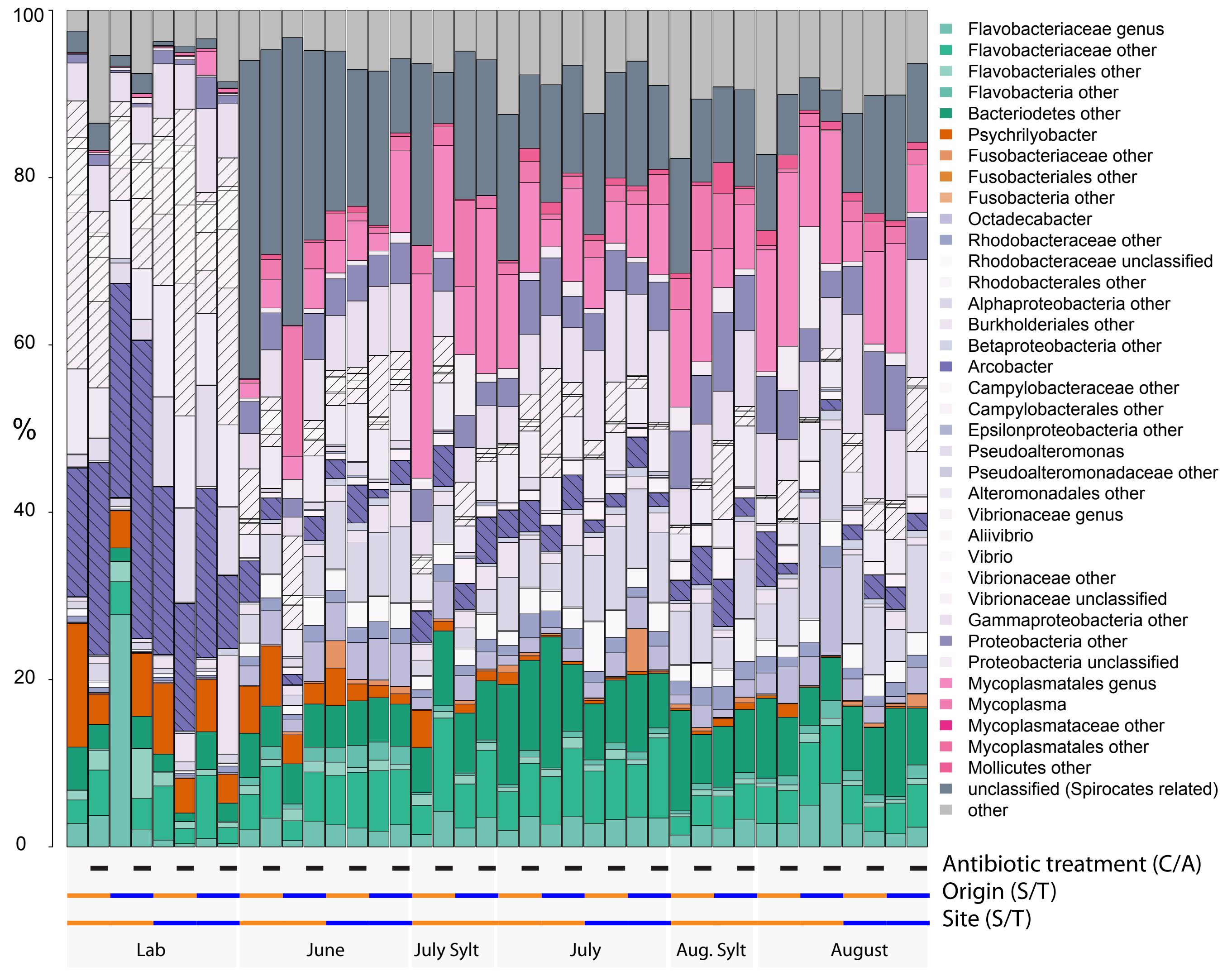


Supplementary Figure S3. Genus level community structure of oyster hemolymph microbiota grouped by date, site, origin and antibiotic treatment. The "unclassifed" bacteria shown in grey mainly consist of a single OTU related to Spirochaetes. Cross-hatching emphasizes the genus *Arcobacter* and genera from *Vibrionaceae*, potentially important oyster symbionts. Only taxa with > 0.1 relative abundance are shown. Note that there are few abundant and fully classified genera. The labels where "other" is added comprise all genera classified within the level escept the ones explicitly named in the legend (i.e. *"Campylobacteriaceae* other" comprises all *Campylobacteriaceae* genera except *Arcobacter*).

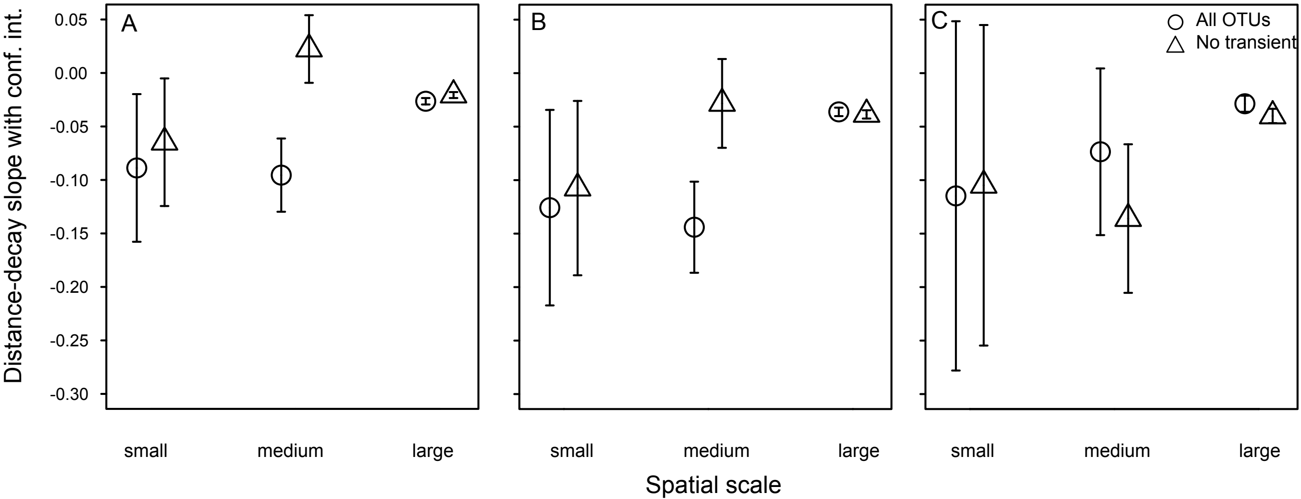


Supplementary Figure S4. Effect of transient OTUs on slope of distance-decay relationship on small, medium and overall spatial scale in A) june. B) july and C) august.

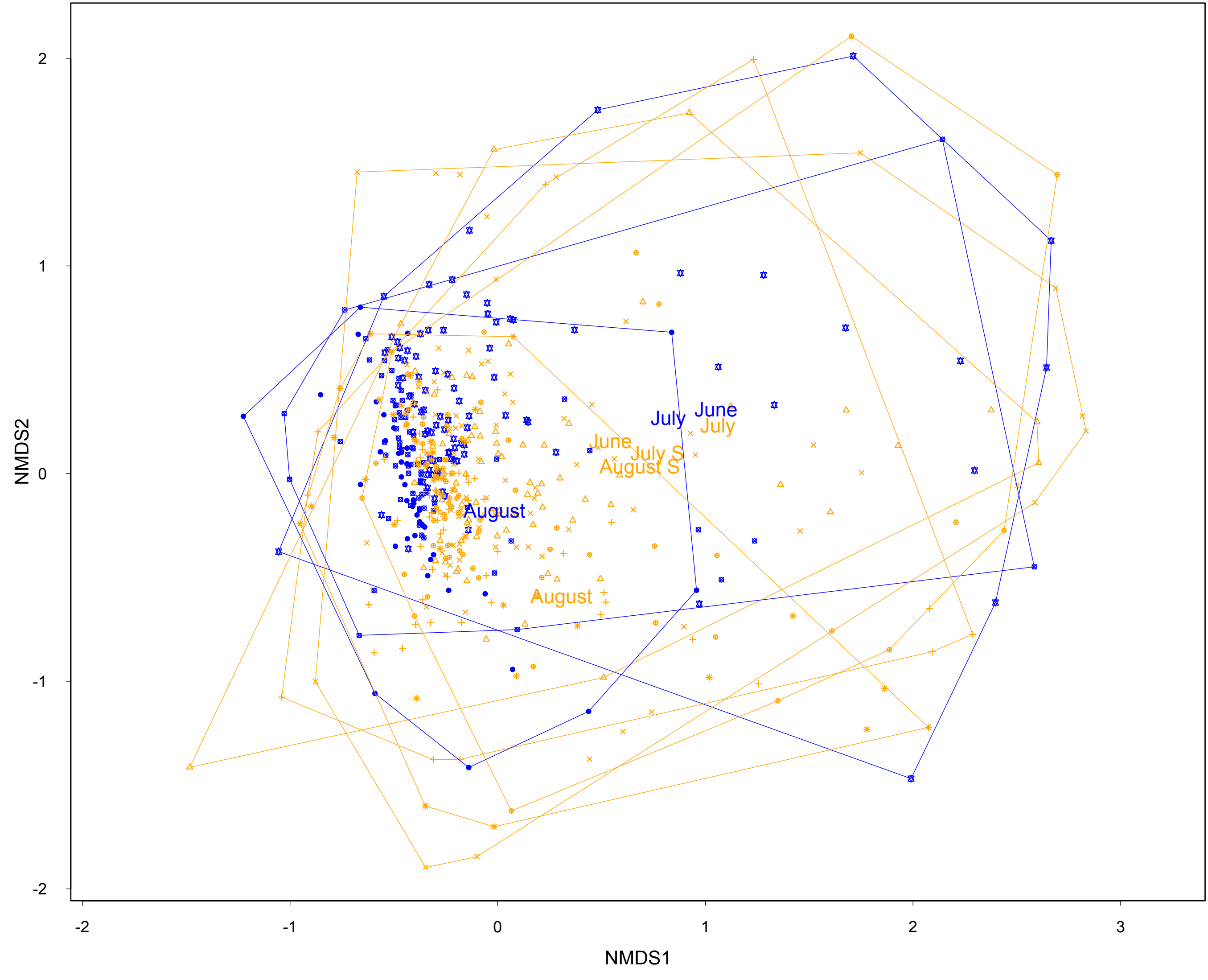


Supplementary Figure S5. NMDS plot (Bray-Curtis) depicting variability of hemolymph microbiota in the field. Labels are placed at the centroid of a given group (site x time, Sylt = orange, Texel =blue). Hulls enclose all the samples from particular site x time combinations.

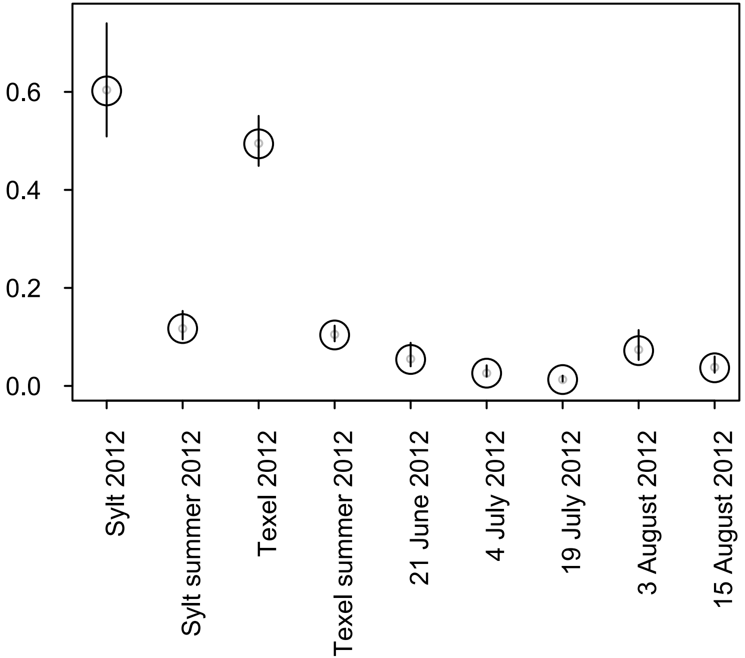


Supplementary Figure S6. Coefficient of variation of all-year temperature measurements, as well as of summer summer months for Texel and Sylt. Last five points are coefficients of variations of measurements took during sampling at individual spots at the same sampling date.
